# Attenuation of Cellular Senescence and Improvement of Osteogenic Differentiation Capacity of Human Liver Stem Cells Using Specific Senomorphic and Senolytic Agents

**DOI:** 10.1007/s12015-025-10876-x

**Published:** 2025-04-12

**Authors:** Allancer D. C. Nunes, Louise E. Pitcher, Henry A. Exner, Diego J. Grassi, Brittan Burns, Maria Beatriz Herrera Sanchez, Ciro Tetta, Giovanni Camussi, Paul D. Robbins

**Affiliations:** 1 Masonic Institute on the Biology of Aging and Metabolism, University of Minnesota, Minneapolis, Minnesota USA; 2 Aelis Farma, Bordeaux, France; 3 Molecular Biotechnology Centre, University of Torino, Torino, Italy; 4 2i3T Societ Per la Gestione Dell’incubatore di Imprese e per il Trasferimento Tecnologico Scarl, University of Torino, Torino, Italy; 5 Unicyte AG, Oberdorf, Switzerland; 6 Department of Medical Sciences, University of Torino, Torino, Italy

**Keywords:** Human liver stem cells, Senotherapeutics, Cellular senescence, Osteogenic differentiation

## Abstract

Expansion of adult stem cells in culture increases the percent of senescent cells, reduces their differentiation capacity and limits their clinical use. Here, we investigated whether treatment with certain senotherapeutic drugs would reduce the accumulation of senescent cells during expansion of human liver stem cells (HLSCs) while maintaining their differentiation capacity. Our results demonstrate that chronic treatment with the senomorphic XJB-5-131 or the senolytics cocktail D + Q reduced the number of senescent cells and significantly reduced the expression of senescence-associated genes and several inflammatory SASP factors in later passage HLSCs. Additionally, treatment with XJB-5-131 and D + Q improved the capacity of HLSCs to undergo osteogenic differentiation following extensive in vitro expansion. Overall, our data demonstrate that treatment with XJB-5-13 or D + Q results in a reduction in the percentage of replication-induced senescent HLSCs and likely other types of adult stem cells and improve the potential therapeutic use of later passage human stem cells.

**Graphical abstract:**

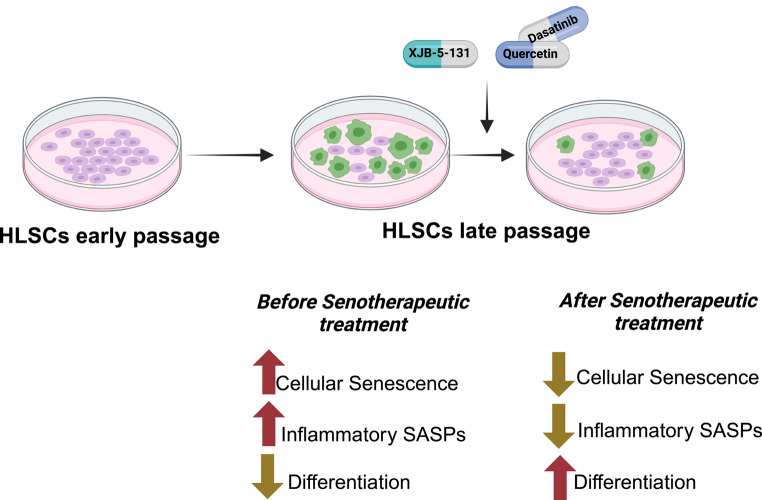

**Supplementary Information:**

The online version contains supplementary material available at 10.1007/s12015-025-10876-x.

## Introduction

Aging is the most important risk factors for many chronic diseases. The prevalence and impact of age-dependent diseases, such as cardiovascular disease, cancer, dementia, diabetes, osteoporosis, renal failure, frailty, and osteoarthritis, are exacerbated with increasing human lifespan [1]. Over 90% of individuals older than 65 years of age have at least one chronic disease, while over 70% have at least two such conditions [2, 3]. The mechanisms thought to contribute, at least in part, to aging including cellular senescence, loss of proteostasis, mitochondrial dysfunction, altered nutrient sensing, telomere attrition, genomic instability, stem cell exhaustion, epigenetic alteration, altered intercellular communication and others are collectively known has the hallmarks of aging [4]. These hallmarks of aging are potential therapeutic targets for healthy aging. Among them, cellular senescence has been demonstrated to be a key driver of aging and age-related diseases and a druggable therapeutic target [5].

Cellular senescence can be induced in response to a variety of stress stimuli, including various types of DNA damage, telomere attrition, nutrient deprivation, hypoxia, oncogene activation, and mitochondrial dysfunction [6, 7]. Senescent cells (SnCs) are broadly characterized by a state of persistent cell cycle arrest, morphological changes, metabolic adaptations, accumulation of damaged macromolecules, genomic instability and upregulation of anti-apoptotic pathways [6]. However, one of the most prominent features of cellular senescence is the senescence-associated secretory phenotype (SASP), defined as the active secretion of pro-inflammatory cytokines, chemokines and interleukins, along with angiogenic factors, cell-growth modulators, matrix-remodeling enzymes, proteases and their inhibitors [8]. Through these SASPs, SnCs modify their environment and influence physiologic and disease processes, impacting stem cell function [6, 8].

Adult stem cell populations play central roles in maintaining tissue homeostasis, promoting repair and, in some cases, regeneration following damage. Based on their self-renewal abilities and capability to develop into various functional cells under certain conditions, stem cell-based therapies have attracted great interest over the past decade [9]. They have been implicated as potential treatments for a variety of conditions, such as neurological, immunological, vascular, cardiac, and renal diseases [9–12]. Despite their therapeutic capacity, stem cells accumulate damage with age, leading to dysfunction with reduced repair and regenerative capacities [13]. In addition, the clinical use of primary stem cells requires expansion in culture to obtain a large number of cells, which often leads to an increase in the percent of senescent stem cells. We previously reported that the injection of stem cells isolated from young, but not old, mice into progeroid mice extends the health span and lifespan through a cell non-autonomous mechanism [14]. Conversely, injections of p16^INK4a^-positive cells into young mice damages cartilage and participates in the progression of osteoarthritis [15].

Several studies have demonstrated the therapeutic benefit of utilizing natural or synthetic compounds that specifically target SnCs, termed senotherapeutics, in alleviating phenotypes associated with age-related chronic disease. Senotherapeutics consist of two classes of compounds: senolytics, which selectively eliminate SnCs (senolysis), and senomorphics, which suppress the SASP without inducing cell death [5]. The first reported and one of the most studied senolytics is the combination of dasatinib (D), a tyrosine kinase inhibitor and FDA-approved anticancer drug, and quercetin (Q), a natural flavonoid [16]. D + Q has shown beneficial effects on bone loss [17], disc degeneration [18], myogenic progenitor cell proliferation [19], and improvement of physical condition and lifespan in mice [20]. Currently, D + Q has been or is currently being tested in numerous human clinical trials for Alzheimer’s disease [21], idiopathic pulmonary fibrosis [22], chronic kidney disease [23], cancer survivors and more. We also previously identified HSP90 inhibitors (e.g., DMAG, 17-AAG), the flavonoid fisetin and Bcl-2 family inhibitors (e.g., Navitoclax, A1331852 and A1155463) as senolytic agents both in culture and in vivo [24].

Oxidative damage and mitochondrial dysfunction are also implicated in aging, stem cell dysfunction, and cellular senescence with antioxidant compounds having senomorphic activity, able to improve stem cell function. In addition, targeting of certain antioxidants to the mitochondria appears to increase their therapeutic effects. For example, XJB-5-131 is a bifunctional antioxidant comprised of the radical scavenger and superoxide dismutase mimetic, 4-hydroxy-2,2,6,6-tetramethyl piperidine-1-oxyl nitroxide (TEMPOL), conjugated to an alkene peptide isostere modification of the Leu-D-Phe-Pro-Val-Orn segment of the antibiotic gramicidin S that allows localization to the mitochondrial membrane [25–27]. XJB-5-131 has been demonstrated to reduce apoptosis, improve mitochondrial function, reduce oxidative damage to mitochondrial DNA, enhance survival in mouse embryonic cells and block cardiolipin oxidation more effectively than TEMPOL [25]. It also reduces brain damage after injury and suppresses motor decline and weight loss in a mouse model of Huntington’s disease [26] while attenuating oxidative DNA damage and senescence in a mouse model of progeria [28].

Here, we investigated whether treatment with the senolytics D + Q, 17-DMAG, fisetin, navitoclax, and the senomorphic mitochondria-targeted free radical scavenger XJB-5-131 could reduce senescent cell accumulation during in vitro expansion of human liver stem cells (HLSCs) and improve their osteogenic differentiation capacity in later passage. The identification of a compound allowing extended expansion of functional stem cells would improve their clinical efficacy by not only improving functional cellular expansion, but also improving the yield of therapeutic stem cell-derived extracellular vesicles (EVs). We demonstrate that treatment with the mitochondrial-targeted radical scavenger XJB-5-131 or the combination of D + Q was sufficient to significantly suppress senescence markers, such as SA-β-Gal and the expression of SASP factors (e.g., *IL-8*, *IL-6 *and *IL-1β*) in replication stress induced senescent HSLCs and improved their osteogenic differentiation capacity in vitro. These novel results demonstrate that the reduction of the senescent stem cell burden is an effective strategy to reduce stem cell dysfunction in later-passage human stem cells.

## Materials and Methods

### Cell Culture and Senotherapeutic Treatment

Human liver stem cells (HLSCs) were isolated from human cryopreserved normal adult hepatocytes (Lonza, Basel, Switzerland, product code: CC-2591). For a more detailed explanation and characterization of the cell line, see [29]. The cells were cultured in αMEM without nucleosides or glutamine (Catalog #: M34450, R&D), supplemented with 10% FBS, 1% penicillin/streptomycin (Gibco), 4 ng/ml basic fibroblast growth factor (FGF) (Catalog #: 170-076-107, Miltenyi Biotec) and epithelial growth factor (EGF) (Catalog #: 170–076-406, Miltenyi Biotec). The cells were maintained at 37 °C and 5% CO_2_. HLSCs were passaged up to passage 16, starting at passage 3. Passaging was performed at 70%-80% confluency. At passage 6, the cells were divided into two different groups. In the first group, HLSCs were chronically treated with the antioxidant compound XJB-5-131 (100 nM) for 24 h every passage until passage 16. For the control group, normal growth media supplemented with 0.1% DMSO was used, and cells were collected for analysis at passage 11 and passage 16. When untreated HLSCs reached passage 11 and passage 16, the cells were divided into two different groups: the control group and the group acutely treated with a combination of dasatinib (D) and quercetin (Q) at 100 nM and 15 μM respectively (D + Q). The cells were collected for analysis after 24 h of D + Q treatment. The DMSO concentration was maintained at 0.1%, and the same concentration of DMSO was used to treat the control group. Other senolytics, such as 17-DMAG (100 nM), fisetin (15 μM), and navitoclax (5 μM), were also tested in HLSCs at passage 11 and passage 16. Since this study does not include clinical or animal experiments, no further ethical approval was required for this research.

### SA-β-gal Senescence Assay

Senescence-associated β-galactosidase was tested using the C12FDG substrate, which becomes fluorescent when hydrolyzed by SA-β-galactosidase. HLSCs treated with XJB-5-131 and D + Q at passages 11 and 16 were plated in 96-well black wall clear bottom plates (Corning) in triplicate at a density of 2500 cells/well. Following the addition of XJB-5-131 (100 nM), D + Q (100 nM and 15 μM) or the control, the cells were incubated for 24 h at 37 °C and 5% CO2. After removing the medium, the cells were incubated with bafilomycin A1 (100 nM) in culture medium for 1 h to induce lysosomal alkalinization, followed by incubation with 20 μM fluorogenic substrate C12FDG (Cayman Chemical) for 2 h and counterstaining with 2 μg/ml Hoechst 33342 (Invitrogen) for 15 min. Finally, the cells were washed with PBS and imaged in six fields per well using the high-content fluorescence image acquisition and analysis platform Cytation 1 (BioTek, USA).

### Immunofluorescence Analysis of Senescent HLSCs

HLSCs at passages 11 and 16 were seeded in 96-well black wall clear glass bottom plates (Povair Sciences) in triplicate at a density of 5000 cells/well. Following the addition of XJB-5-131 (100 nM), D + Q (100 nM and 15 μM) or the control, the cells were incubated for 24 h at 37 °C and 5% CO_2_. Subsequently, HLSCs were fixed with 4% paraformaldehyde for 10 min at room temperature and permeabilized with 1% Triton X-100 in PBS for 10 min at room temperature. Next, the cells were blocked with 10% normal goal serum (Invitrogen) and incubated with anti-γH2AX primary antibody (Cell Signaling Technology, 9718S; 1:1000) overnight at 4 °C. After incubation with primary antibody, the cells were washed three times with PBS and incubated with Alexa Fluor 647-conjugated anti-rabbit secondary antibody (Abcam, ab150077) at a 1:2000 dilution for 2 h at room temperature, followed by three more washes with PBS. Finally, the cells were counterstained with Hoechst 33342 (Invitrogen) at a 1:1000 dilution for 30 min at room temperature. Images were acquired using the high content fluorescence image acquisition and analysis platform Cytation 1 (BioTek).

### RNA Extraction and qRT‒PCR

Total RNA was isolated from HLSCs using QIAzol, followed by RNA extraction using the miRNeasy Mini Kit (Qiagen, The Netherlands) according to the manufacturer’s protocol. The isolated RNA was quantified using a NanoDrop spectrophotometer (Thermo Fisher, USA) and stored at -80 °C until further use. For the synthesis of cDNA, 400 ng of RNA was reverse transcribed using an iScript cDNA synthesis kit (Bio-Rad, USA) according to the manufacturer’s protocol. Reactions were set up in a MicroAmp Fast Optical 96-well reaction plate with 2 μL of 1:5 diluted cDNA, 0.2 μL of specific oligonucleotide primers (Table S1), 12.6 μL of nuclease-free water, and 5 μL of Fast SYBR Green Master Mix (Applied Biosystems, USA)/well. β-2-microglobulin (B2M) served as a housekeeping gene. The data were analyzed using the 2-ΔΔCt method.

### Bulk RNA Sequencing Analysis

HLSCs at early-passage HLSCs (passage 6) or passage 11 and passage 16 treated with XJB-5-131, D + Q or DMSO control were randomly selected for bulk RNA sequencing. Library preparation and sequencing was performed at the University of Minnesota Genomics Center. Unique dual indexed paired-end TruSeq stranded libraries were created and pooled following rRNA depletion using the Ribo-Zero Plus rRNA Depletion kit. All libraries were gel size selected ($\approx$ 200 bp) and sequenced using an Illumina NovaSeq X Lane with a 2 × 150 bp flow cell. Libraries were sequenced to a mean depth of $\ge$ 20 M reads. Fastqc files were processed using Trimmomatic for the removal of adapter sequences and bases having a Q-score < 30. The quality of trimmed fastqc files were confirmed using FastQC. Paired-end reads were aligned to the GRCh38 reference genome using Hisat2 v2.0.5 and feature Counts v1.5.0 was used count and map each gene. Pre-filtering removing low count reads ($\le 5$) and Differential Gene expression (DEG) analysis was done in R v4.1.0 using the package DESeq2 v3.19 and a false discovery rate cut off of 0.05. Ensemble IDs were converted to HGNC IDs using the R package biomaRT. Differentially expressed genes with adjusted *p*-values < 0.05 and |log2FC|> 2 were selected for downstream over representation analysis (ORA) using the Database for Annotation, Visualization and Integrated Discovery (DAVID) and the Kyoto Encyclopedia of Genes and Genomes (KEGG) database.

### In Vitro Differentiation of HLSCs

Osteogenic differentiation was performed with the Human Mesenchymal Stem Cell (hMSC) Osteogenic Differentiation Medium BulletKit (Lonza, Italy). HLSCs were seeded at an initial density of 1 × 10^5^ cells/well in a 12-well plate and cultured in osteogenic induction medium for a total of 14 days, with the medium changed every 3 days. The calcification matrix was visualized with Alizarin Red after osteogenic induction. A blank group without differentiation induction was included. Calcium deposits were observed with an EVOS FL Auto Cell Imaging System (Thermo Fisher, USA) photomicroscope at 200X magnification. An osteogenesis quantification kit (Millipore) was used for the quantification of calcium deposits. After alizarin red staining, 10% acetic acid was added to collect the cells, which were then incubated at 85 °C for 10 min and transferred to ice for 5 min. The samples were centrifuged at 20,000 × g for 15 min. Subsequently, the supernatant was transferred to a new tube, and the pH was adjusted to 4.1–4.5 using 10% ammonium hydroxide. The absorbance was measured at 405 nm using a Varioskan LUX plate reader (Thermo Fisher, USA).

### Statistical Analysis

Data analyses were performed using GraphPad Prism 10. The results are generally expressed as the mean ± SEM. Statistical analysis was performed by ANOVA with Tukey’s multiple comparison test. A p value < 0.05 was considered significant.

## Results

### Accumulation of Senescent Cells during Human Liver Stem Cell Expansion

**Fig. 1:**
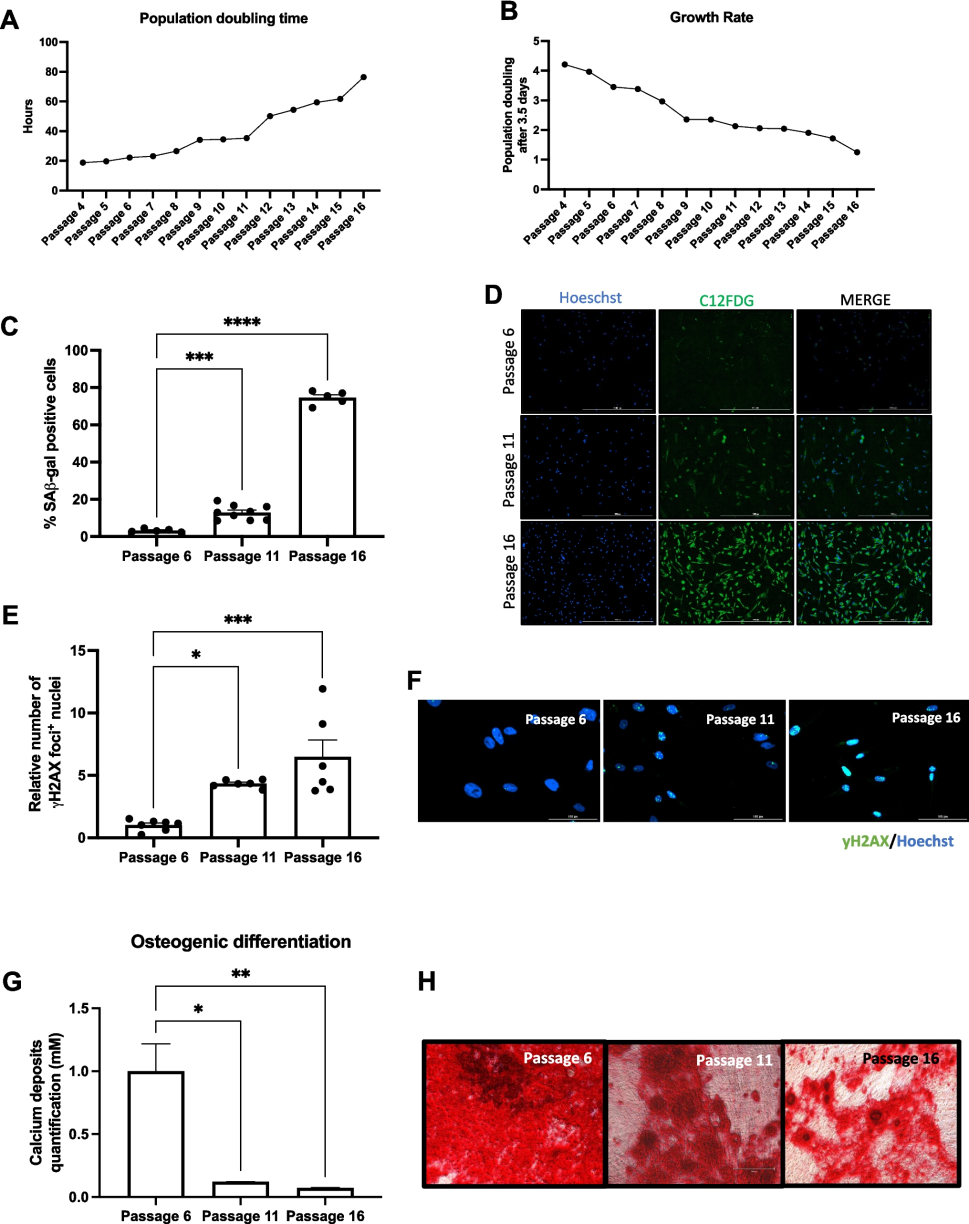
Accumulation of cellular senescence during HLSC expansion. **A** and **B** Population doubling time and growth rate of HLSCs during expansion. **C** and **D** Quantification and representative images of SA-β-Gal-positive HLSCs at passages 6, 11 and 16. **E** and **F** Quantification and representative immunofluorescence images of γH2AX-positive HLSCs at passages 6, 11 and 16. **G** and **H** Quantification of calcium deposits and representative alizarin red images following 14 days of osteogenic differentiation. All the data are shown as means ± SEM. *p < 0.05, **p < 0.01, ***p < 0.001 and ****p < 0.0001

Propagation of primary mammalian adult stem cells results in an increase in the percentage of cells expressing markers of senescence and a reduction in the rate of proliferation. In turn, this reduces their function and thus their therapeutic effect. We observed that continuous expansion of HLSCs increased the population doubling time and decreased the growth rate (Fig. 1A, B). Using the fluorescent substrate C12FDG to quantify the number of cells positive for senescence-associated β-galactosidase (SA-β-Gal) activity, we also demonstrated that there was an increase in the percentage of senescent HLSCs with passage: 13% at passage 11 and 74.5% at passage 16 compared to 3.2% at early passage 6 (Fig. 1C, D). Since replication stress is known to increase DNA damage, which can drive cells into a senescent state, the percent of cells exhibiting persistent γ-H2AX foci, a marker of DNA damage, was determined. We observed an increase of 4- and 6.5-fold in the percent of HLSCs at passage 11 and 16, respectively, that were γ-H2AX-positive foci (Fig. 1E, F).

To determine if the accumulation of SnCs impacts the capacity of stem cells to differentiate during in vitro expansion, we induced osteogenic differentiation using late-passage HLSCs and quantified calcium deposition by alizarin red staining. Consistent with the reduction in proliferation, increased DNA damage and senescence, HLSCs at passage 11 and 16 had reduced osteogenic activity (Fig. 1G, H). These results show that extended in vitro expansion of adult human stem cells increases the presence of senescence markers and reduces their ability to undergo osteogenic differentiation.

### XJB-5-131 and D + Q Treatment Attenuates Cellular Senescence in Late Passage HLSCs

**Fig. 2:**
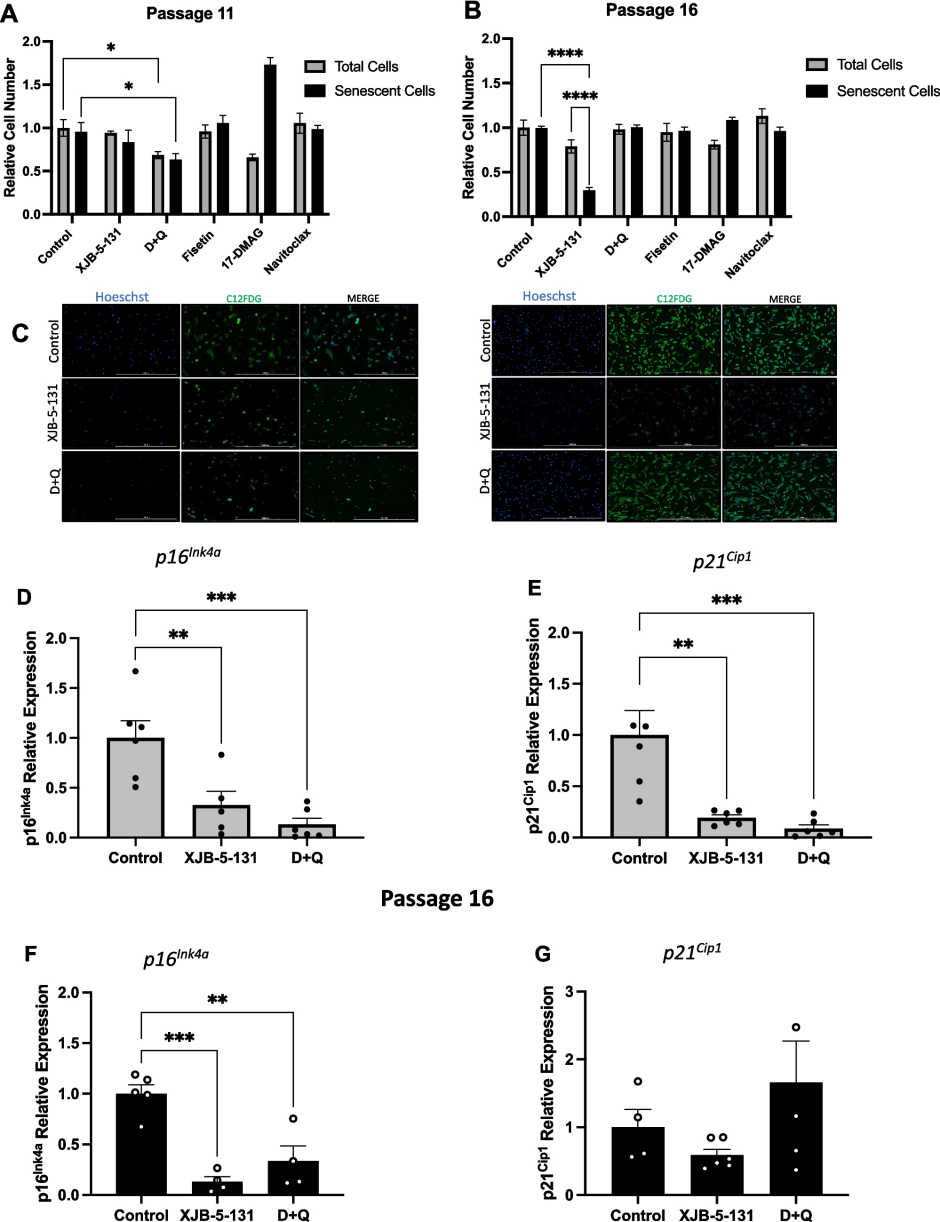
XJB-5-131 and D + Q reduced the expression of senescence markers in late-passage HLSCs. **A** to **C** Quantification and representative images of SA-β-Gal-positive HLSCs at passages 11 and 16 after treatment with XJB-5-131, D + Q, Fisetin, 17-DMAG and Navitoclax. **D** to **G** Expression of the senescence markers p16^INK4a^ and p21^CIP1^ in HLSCs at passages 11 and 16 after treatment with XJB-5-131 and D + Q. All the data are shown as means ± SEM. **p < 0.01, ***p < 0.001 and ****p < 0.0001

Given the increase in the percent of SnCs during the expansion of HLSCs in culture, we hypothesized that SnC removal and/or SASP suppression during expansion would lead to an improved functionality in the remaining stem cells. Therefore, we examined the potential benefit of chronic or acute treatment with a senomorphic (XJB-5-131), a mitochondrial targeted free radical scavenger, and four senolytics (D + Q, fisetin, 17-DMAG and navitoclax) in eliminating SnCs and/or suppressing the SASPs. Following chronic treatment with XJB-5-131 in HLSCs we observed a reduction in the number of SnCs without changing the total number of cells at passage 16, suggesting this compound was working as a senomorphic, suppressing expression of senescent cell markers (Fig. 2B, C). Following acute treatment with the senolytic combination of D + Q, fisetin, 17-DMAG or navitoclax, we observed a significant reduction in the total number of cells and the number of SnCs after treatment with D + Q at passage 11, consistent with a senolytic effect of D + Q (Fig. 2A and C). However, we did not observe any senolytic effects using 17-DMAG, fisetin, or navitoclax in HLSCs at either passage (Fig. 2A-B and Fig. S1A-B). In fact, 17-DMAG increased the percent of SA-ß-gal positive HLSCs at passage 11.

To examine further any potential senotherapeutic effects of XJB-5-131, D + Q, fisetin, 17-DMAG and navitoclax on high passaged HLSCs, the expression of senescence markers associated with cell cycle arrest (p16^INK4a^ and p21^Cip1^) was quantified by RT‒qPCR. After treatment, XJB-5-131 and D + Q strongly reduced the expression of *p16*^*INK4a*^ and *p21*^*Cip1*^ in HLSCs at passage 11 (Fig. 2D, E). At passage 16, only *p16*^*INK4a*^ was downregulated after XJB-5-131 and D + Q treatment (Fig. 2F). In contrast, no effect on *p21*^*Cip1*^ expression was observed in HLSC passage 16 (Fig. 2G). In addition, no effects on *p16*^*INK4a*^ and *p21*^*Cip1*^ expression were observed after 17-DMAG, fisetin or navitoclax treatment (Fig. S1C-F).

**Fig. 3:**
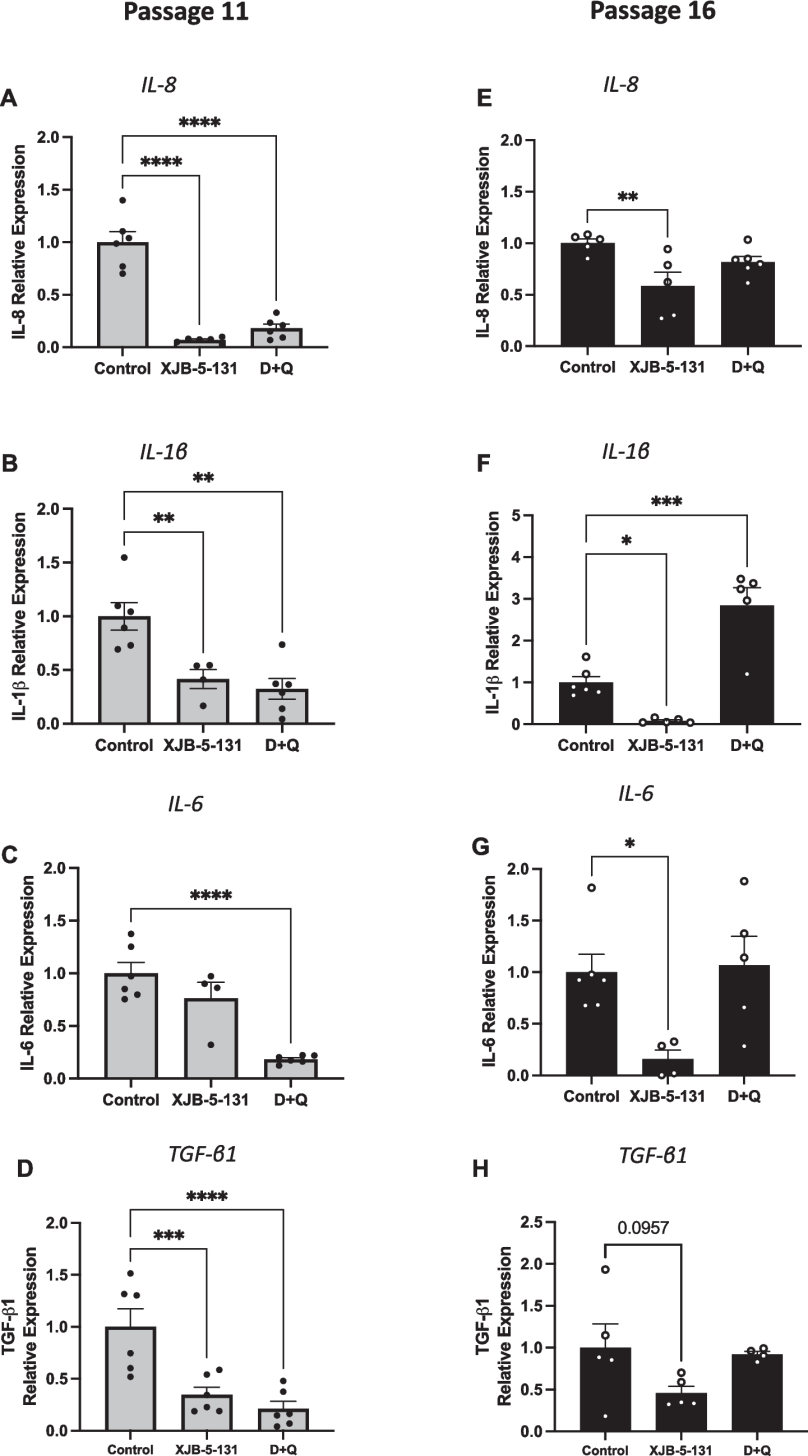
XJB-5-131 and D + Q downregulated SASP expression in later-passage HLSCs. **A** to **D** Quantification of the SASP factors IL-8, IL-1β, IL-6 and TGF-β1 in HLSCs at passage 11 after treatment with XJB-5-131 and D + Q. **E** to **F** Quantification of the SASP factors IL-8, IL-1β, IL-6 and TGF-β1 in HLSCs at passage 16 after treatment with XJB-5-131 and D + Q. All the data are shown as means ± SEM. *p < 0.05, **p < 0.01, ***p < 0.001 and ****p < 0.0001

As SnCs secrete many SASP factors, we also evaluated the expression of several of the proinflammatory cytokine/chemokine SASP factors by qPCR. As shown in Fig. 3, treatment with XJB-5-131 and D + Q significantly reduced the expression of *IL-8*, *IL-1β *and *TGF-β1* in HLSCs at passage 11 (Fig. 3A, B and D). *IL-6* was downregulated only after D + Q treatment at passage 11 (Fig. 3C). Moreover, chronic treatment with XJB-5-131 more efficiently reduced the expression of SASPs in HLSCs at passage 16, decreasing the expression of *IL-8*, *IL-1β*, *IL-6* and *TGF-β1* (Fig. 3E-H). Overall, these findings suggest that treatment with the senotherapeutics XJB-5-131 and D + Q reduce replication stress-induced senescence in HLSCs or eliminate senescent HLSCs respectively and therefore we examined the effects of these two compounds in further detail.

### XJB-5-131 and D + Q Treatment Downregulates SASP Factors Following Long-Term Culturing of HLSCs

**Fig. 4:**
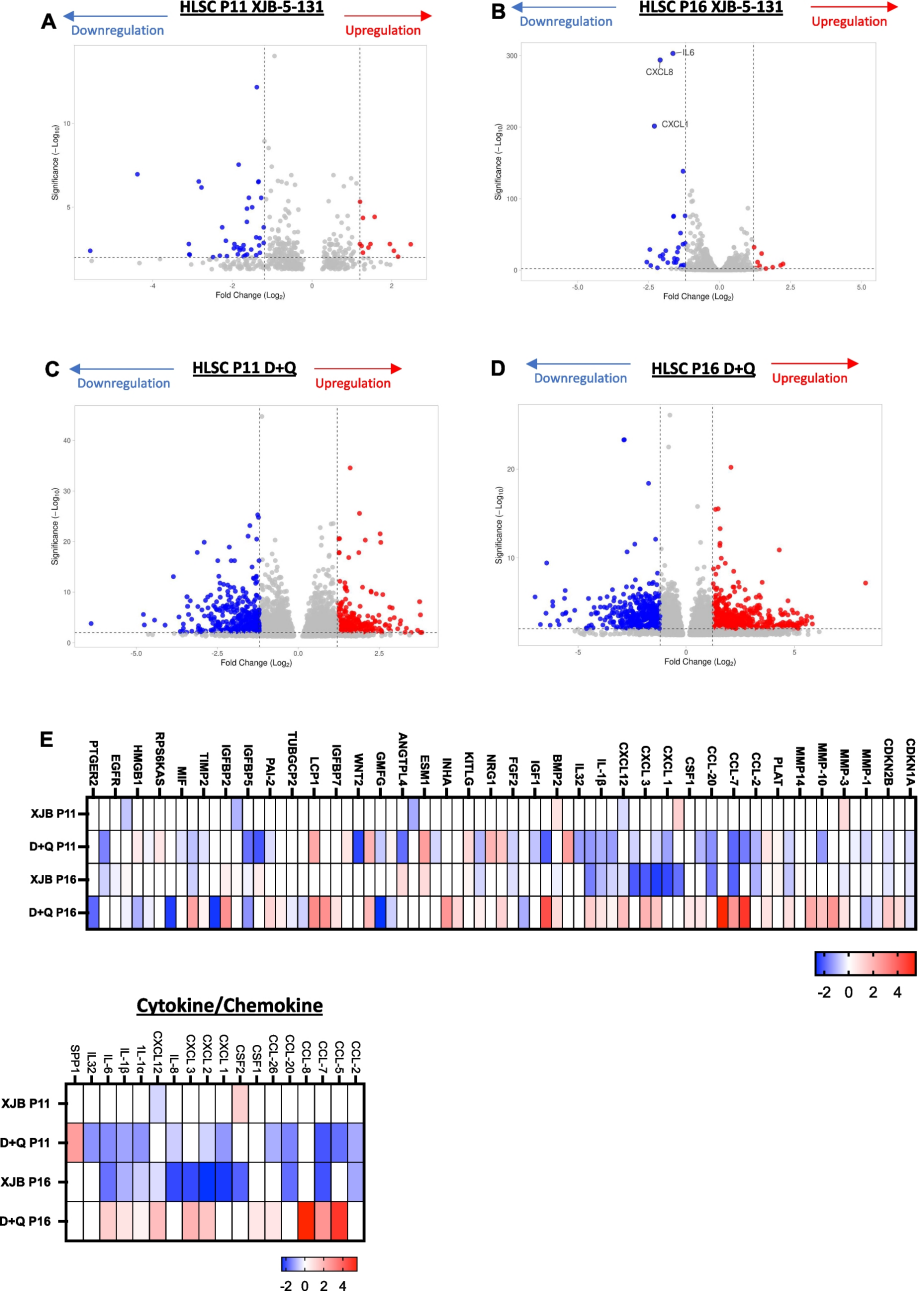
Downregulation of SASPs from the SenMayo panel induced by XJB-5-131 and D + Q treatment in HLSCs at passages 11 and 16. **A** to **D** Volcano plot showing the distribution of downregulated and upregulated genes after XJB-5-131 and D + Q treatment in HLSCs at passages 11 and 16. **E** Heatmap of SASP genes from the SenMayo panel showing statistically significant differences between HLSCs at passages 11 and 16 treated with XJB-5-131 or D + Q and control HLSCs at passage 11 or 16

To further explore the senotherapeutic activity of XJB-5-131 and D + Q treatment on late-passage adult human stem cells, we performed bulk RNA-seq analysis of HLSCs at passage 11 and passage 16. Analysis of differentially expressed genes showed that XJB-5-131 treatment at passage 11 had no significant transcriptional impact when compared to the control (Fig. 4A). However, the same treatment significantly downregulated the expression of important cytokines/chemokines, such as *IL-6*, *CXCL8* and *CXCL1* in HLSCs at passage 16 (Fig. 4B). In addition, D + Q treatment downregulated and upregulated several genes, including genes encoding multiple SASP factors, in both groups of cells (Fig. 4C, D). Consistent with the RT‒qPCR data, we observed a significant overall reduction in the expression of SASP factors, mostly cytokines/chemokines, in HLSCs at passage 11 treated with D + Q and in HLSCs at passage 16 treated with XJB-5-131 (Fig. 4E).

Over representation analysis of KEGG pathways revealed that both treatments significantly downregulated inflammatory pathways, such as cytokine‒cytokine receptor interaction as well as the TNF and chemokine signaling pathways (Fig. S2A). Inflammatory responses were the most impacted biological process downregulated in HLSCs at passage 11 treated with D + Q and in HLSCs passage p16 treated with XJB-5-131 (Fig. S2B). Taken together, these data suggest that XJB-5-131 and D + Q decrease SASP expression at different time points after HLSC expansion, reducing senescence in adult human stem cells during long term culture.

### D + Q Treatment Upregulated DNA Damage Repair-related Genes in Late Passage HLSCs

**Fig. 5:**
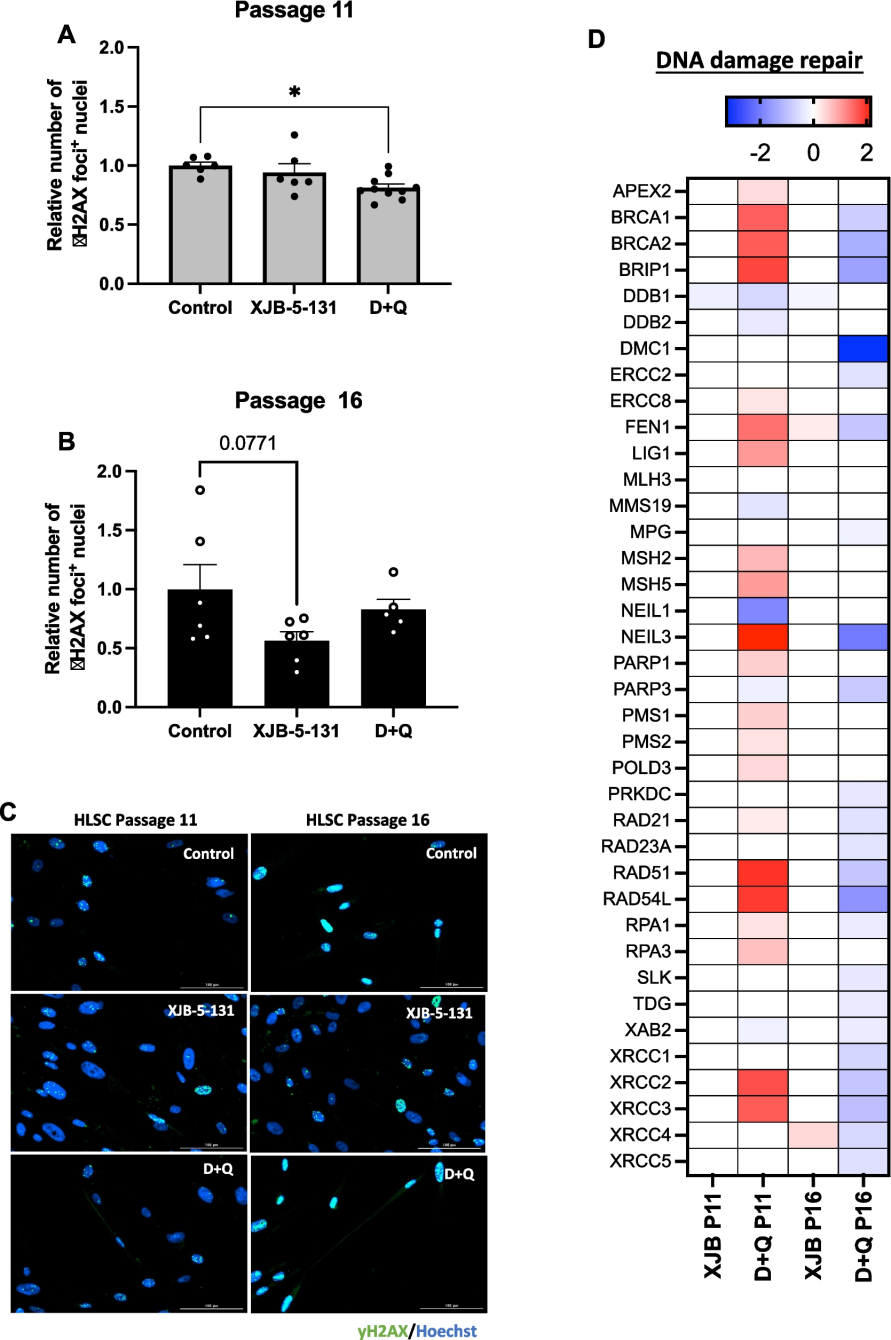
D + Q treatment decreased γH2AX foci and upregulated relevant DNA damage repair-related genes. **A** to **C** Quantification and representative immunofluorescence images of γH2AX foci + nuclei in HLSCs at passages 11 and 16 after treatment with XJB-5-131 and D + Q. **D** Heatmap of DNA damage repair-related genes significantly different between HLSCs at passages 11 and 16 treated with XJB-5-131 or D + Q and control HLSCs at passage 11 or 16. All the data are shown as means ± SEM. *p < 0.05

Cellular senescence is often triggered by irreparable DNA damage, such as double stranded DNA breaks and telomere attrition, as a consequence of proliferation, which accumulates during the extensive in vitro expansion. Here, we quantified γH2AX foci-positive cells and analyzed the expression of several DNA damage repair-related genes in HLSCs at passages 11 and 16 after treatment with XJB-5-131 and D + Q. As shown in Fig. 5A and C, only D + Q significantly decreased the number of γH2AX-positive cells in HLSCs at passage 11. In contrast, in HLSCs at passage 16, only XJB-5-131 slightly decreased the number of γH2AX foci-positive cells (Fig. 5B and C). Based on these results, we examined the expression of several DNA damage repair-related genes and observed that D + Q significantly upregulated the expression of several genes important for DNA damage repair, e.g., BRIP1, RAD51 and NEIL3, at passage 11 (Fig. 5D). Interestingly, D + Q induced the opposite effect in HLSCs at passage 16, downregulating many DNA damage repair-related genes (Fig. 5D). Overall, these results suggest that in addition to downregulating senescence and SASP factors, treatment with certain senotherapeutics also have the potential to regulate DNA damage and repair in aged adult human stem cells.

### D + Q and XJB-5-131 Improved the Capacity of Late Passage HLSCs to Undergo Osteogenic Differentiation

**Fig. 6:**
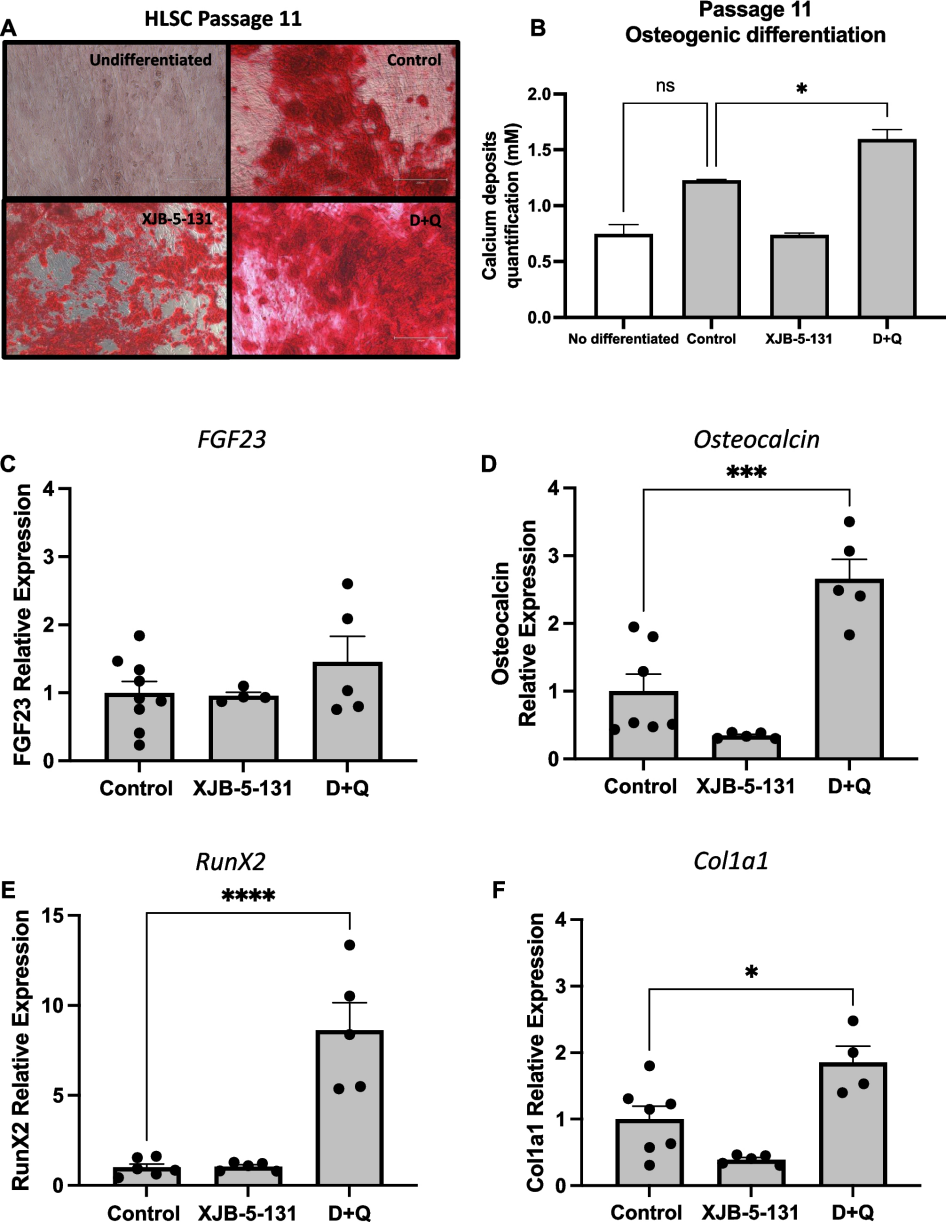
Treatment with D + Q improved osteogenic differentiation of HLSCs at passage 11. **A** and **B** Representative alizarin red images and quantification of calcium deposits following 14 days of osteogenic differentiation of HLSCs at passage 11 after treatment with XJB-5-131 and D + Q. **C** to **F** Quantification of the osteogenic markers *FGF23*, *Osteocalcin*, *RunX2* and *Col1a1* following 14 days of osteogenic differentiation of HLSCs at passage 11 after treatment with XJB-5-131 and D + Q. All the data are shown as means ± SEM. *p < 0.05, ***p < 0.001 and ****p < 0.0001

HLSCs are multipotent and are thus able to differentiate into several types of cells, e.g., hepatocytes, osteocytes, and endothelial cells. To determine whether treatment with D + Q or XJB in late passage could improve HLSC function we investigated the ability of these late passage cells to undergo the osteogenic differentiation. As shown in Fig. 6A, D + Q treatment improved calcium deposition indicated by increased alizarin red intensity and quantification compared to the control (Fig. 6B). Osteogenic differentiation was further confirmed by examining the expression of osteogenic gene markers *FGF23*, *osteocalcin*, *RunX2* and collagen I (*Col1a1*) by RT‒qPCR. *Osteocalcin*, *RunX2* and *Col1a1* were significantly up-regulated in HLSCs at passage 11 treated with D + Q compared with those in the control group after 14 days of differentiation (Fig. 6D-F) whereas there was no difference in *FGF23* expression (Fig. 6C). XJB-5-131 treatment did not improve the expression of these markers in HLSC at passage 11.

**Fig. 7:**
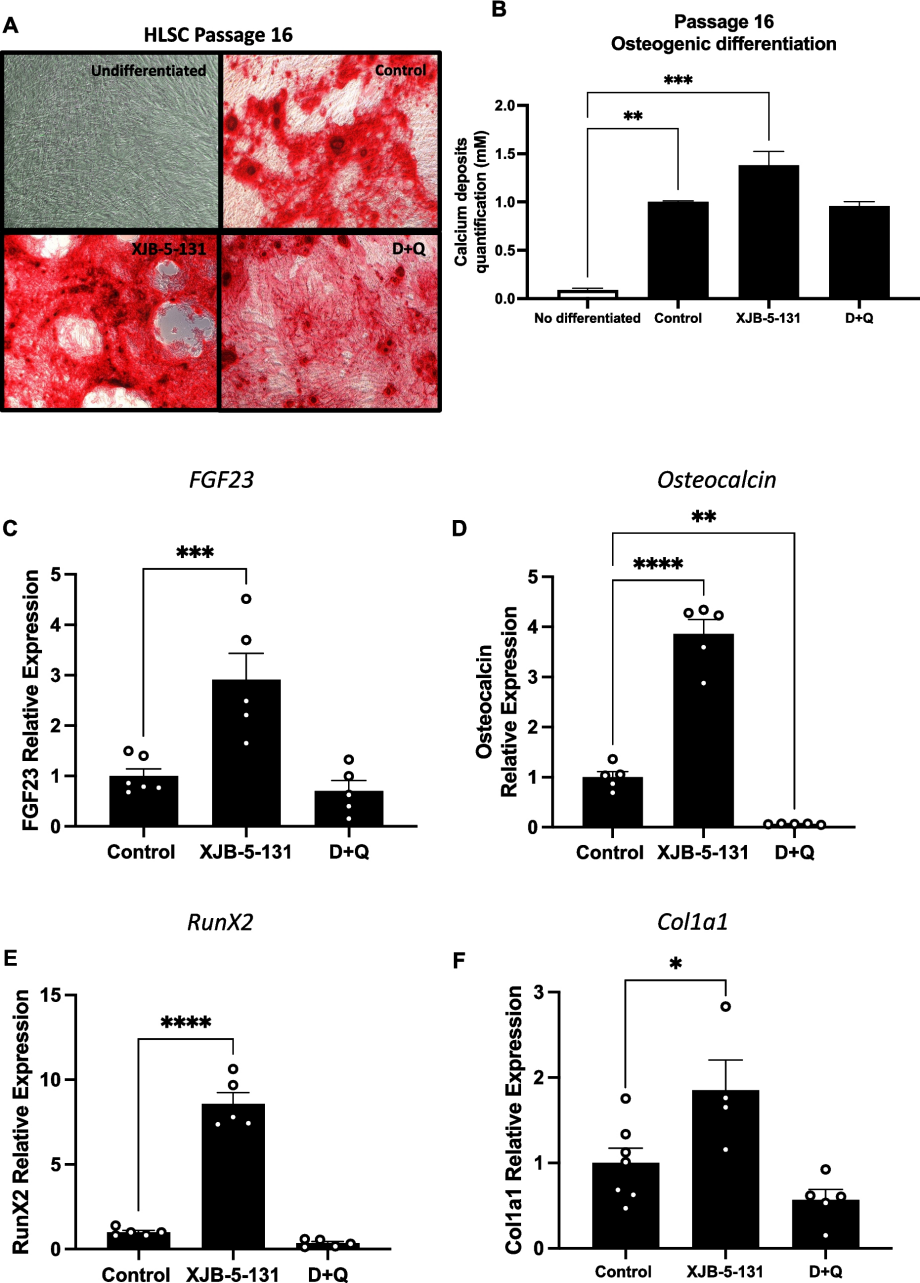
Treatment with XJB-5-131 improved osteogenic differentiation of HLSCs at passage 16. **A** and **B** Representative alizarin red images and quantification of calcium deposits following 14 days of osteogenic differentiation of HLSCs at passage 16 after treatment with XJB-5-131 and D + Q. **C** to **F** Quantification of the osteogenic markers *FGF23*, *Osteocalcin*, *RunX2* and *Col1a1* following 14 days of osteogenic differentiation of HLSCs at passage 16 after treatment with XJB-5-131 and D + Q. All the data are shown as means ± SEM. *p < 0.05, **p < 0.01, ***p < 0.001 and ****p < 0.0001

Given the passage-dependent senotherapeutic effects observed between passages 11 and 16, we also explored the osteogenic differentiation capacity of HLSCs at passage 16. As shown in Fig. 7A, alizarin red staining was greater in HLSC passage 16 treated with XJB-5-131 than in the control group. Notably, only HLSCs at passage 16 treated with XJB-5-131 exhibited a significant increase in deposited calcium compared with those in the control group (Fig. 7B). In addition, the expression of *FGF23*, *osteocalcin*, *RunX2*, and *Col1a1* in HLSCs at passage 16 treated with XJB-5-131 after 14 days of differentiation was significantly greater than that in the control group (Fig. 7C-F). In contrast, the results for passage 16 HLSCs treated with D + Q were not as significant as the results observed for passage 11. In summary, these results show that treatment with XJB-5-131 and D + Q could preserve or improve the differentiation potential of adult human stem cells in a passage-dependent manner.

## Discussion

Senescence is a cellular response phenotype characterized by increased metabolic activity and resistance to apoptotic cell death while remaining in a state of cell cycle arrest that limits the proliferation potential of cells [6]. Primary stem cells are often cultivated for extensive passages to achieve the number of cells required for therapeutic applications. The expansion of stem cells in culture leads to replicative senescence [30], which affects their phenotype and therefore therapeutic efficacy [31, 32].

Like other human stem cells, HLSCs, a stem cell population in the liver also display a progressive loss of stemness during expansion in culture [29]*.* This is likely due to the increase in senescence and secreted SASP factors which induce a state of chronic inflammation leading to a reduction in proliferation and differentiation capacity. Like MSCs, HLSCs also exhibit the multipotency potential to differentiate into several types of cells, such as hepatocytes, pancreatic cells, and cells of osteogenic lineages [29, 33]. Early passage HLSCs are able to undergo osteogenesis, forming calcium deposits and expressing bone-specific proteins, e.g., osteocalcin and osteopontin, after 3 weeks of culture in osteogenic differentiation medium [29]. However, unlike MSCs, no adipogenic differentiation has been reported for HLSCs.

Stem cell-based therapies have significant potential in the field of regenerative medicine, facilitating repair of damaged tissues and organs. Improving the function of adult stem cells that need to be expanded in culture is crucial for their ability to repair tissues in vivo [34]. Stem/progenitor cell-based therapies have been developed for the treatment of vascular, skin and neurodegenerative diseases. However, oxidative stress, chronic inflammation and cellular senescence are the major compromisers of the regenerative potential of stem cells following passage in culture and/or if isolated from older individuals. For example, age-related decline in endothelial colony-forming cell quantity and functionality contributes to vascular rarefaction, diminished cerebral blood flow and blood-brain barrier permeability [35]. Thus, strategies allowing the generation of a large number of functional stem cells with retained stemness, and lineage plasticity are required for clinical applications or for the isolation of therapeutic EVs. Pharmacological approaches have been employed as tools to prevent senescence in culture. For example, human MSCs cultured in the presence of rapamycin showed a high proliferative rate and osteogenic differentiation potential, possibly through the inhibition of the Akt/mTOR pathway [36].

In the present study, we examined the use of the senotherapeutics to eliminate or reverse senescence, improving the clinical application of human stem cells. Our results demonstrate that HLSCs treated with D + Q and XJB-5-131 acquire a reduced senescence profile, characterized by a decrease in the number of SA-β-Gal-positive cells and γH2AX foci-positive nuclei along with a significant downregulation of cyclin-dependent kinase inhibitors p16^INK4a^ and p21^CIP1^ and SASP factors, especially the cytokines/chemokines *IL-8*, *IL-1β* and *IL-6*. We also observed the upregulation of DNA damage repair-related genes. Concomitantly with the reduction in senescence, we find that treatment also improved the osteogenic differentiation capacity of late passage HLSCs.

Among the senotherapeutics we tested, D + Q has shown extensive efficacy in targeting the burden of SnCs and alleviating age-related diseases through functional improvement of vital tissues such as adipose tissue, muscles, brain, kidney and liver [37–41]. We demonstrate that D + Q ameliorated the detrimental effects induced through extensive in vitro expansion of HLSCs by reducing senescence and DNA damage and improving osteogenic differentiation capacity. Consistent with our findings, D + Q was shown to effectively eliminate senescent mouse bone marrow stem cells, improve their osteogenic capacity, and decrease several senescence and SASP factors both in vitro and in vivo [42]. We also observed that the most significant effects of D + Q were in HLSCs at passage 11. A significant clearance of SnCs and improvement in osteogenic capacity were not observed in HLSC at passage 16 after treatment with D + Q. These may be due to the relatively high percentage of senescent HLSCs (74.5%) at passage 16, which affected the efficacy of D + Q on senolysis in vitro, and consequently not restoring their osteogenic differentiation capacity.

An important strategy to target SnCss is the suppression of oxidative stress. The accumulation of ROS during aging and pathological conditions is the fundamental source of oxidative stress that contributes to cell damage and senescence [13, 43, 44]. Numerous studies have reported that the control of ROS is an effective strategy for preventing vascular aging, brain aging, neurodegenerative diseases and extending the survival of stem cells with functional integrity. Pharmacological and non-pharmacological antioxidant strategies, alone or in combination, have shown potential to alleviate senescence, and several are being used for anti-aging studies in human and mouse cells [13, 45–47]. Non- pharmacological interventions, e.g., dietary restriction, remarkably improve mitochondrial function and reduces oxidative stress in the aortas of aged mice [47]. In our study, XJB-5-131, a synthetic mitochondria-targeted free radical scavenger, rescued the osteogenic differentiation capacity and reduced senescence levels of exhaustive expanded HLSCs by significantly suppressing expression of *p16*^*INK4a*^*, p21*^*CIP*^ and many cytokines/chemokines, such as *IL-8*, *IL-1β* and *IL-6*, reducing DNA damage and upregulating several genes involved on DNA damage repair. Similarly, other antioxidants, such as glutathione, melatonin and edaravone (a synthetic free-radical scavenger) have been reported to rescue the function of elderly ADMSCs by reducing ROS levels and the number SA-β-Gal-positive cells [48, 49].

Interestingly, the other senotherapeutic drugs tested, 17-DMAG, Fisetin and Navitoclax, did not induce any senotherapeutic effects in HLSCs at passages 11 or 16. Several studies have demonstrated that the effects of senotherapeutics are cell type dependent and the different stimuli to induce senescence can also affect the activity of senotherapeutics. Navitoclax, an inhibitor of the Bcl-2 family of anti-apoptotic proteins, demonstrated senolytic activity in senescent human umbilical vein endothelial cells (HUVECs) and IMR90 human lung fibroblasts, but not senescent human primary preadipocytes. Fisetin, a member of the flavonoid family found in common foods and available as an oral dietary supplement, did not eliminate radiation-induced senescent IMR90s nor adipose-derived stem cells (ADSCs) [50, 51], but it eliminated etoposide-induced senescent IMR90 and passage-induced senescent ADSCs [51, 52]. Although very potent senolytics, not all HSP90 inhibitors work on all cell types. For example, 17-DMAG significantly reduced senescence in IMR90s and human WI38 fibroblasts, but Ganestespib, another HSP90 inhibitor, is more effective eliminating HUVECs induced to senesce with ionizing radiation, but not preadipocytes [24].

## Conclusion

In summary, here we demonstrated that long-term expansion of HLSCs in culture, and likely all human stem cells, undergo replicative senescence, as demonstrated by a decline in growth rate as well as a significant increase in senescence markers, which was correlated with reduced differentiation capacity. We also demonstrate that intermittent use of D + Q or chronic use of XJB-5-131 on late passage HLSCs is able to eliminate SnCs or attenuate their SASP, which allows for the increased expansion and improved differentiation capabilities of the remaining non-senescent cells. Thus, the use of these senotherapeutic compounds can increase the potential therapeutic efficacy of adult human stem cells undergoing expansion in culture.

## Supplementary Information

Below is the link to the electronic supplementary material.

Supplementary file1 (PDF 3642 KB) Supplemental Figure 1. 17-DMAG, Fisetin and Navitoclax did not reduce senescence in late-passage adult human stem cells. (**A** and **B**) Expression of the senescence markers p16^INK4a^ and p21^CIP1^ in HLSCs at passage 11 after 24 hours treatment with Fisetin, 17-DMAg and Navitoclax. (**C** and **D**) Expression of the senescence markers p16^INK4a^ and p21^CIP1^ in HLSCs at passage 16 after 24 hours treatment with Fisetin, 17-DMAG or Navitoclax. All the data are shown as means ± SEM. *p<0.05, **p<0.01 and ****p<0.0001. Supplemental Figure 2. Analysis of the top 10 KEGG pathways and GO terms. (**A**) Log (*p* value) analysis of the top 10 KEGG pathways in HLSCs at passages 11 and 16 after XJB-5-131 and D+Q treatment. Blue=upregulated and Red=downregulated. (**B**) Log (*p* value) analysis of the top 10 GO terms in HLSCs at passages 11 and 16 after XJB-5-131 and D+Q treatment. Blue=upregulated and Red=downregulated.

Supplementary file2 (DOCX 16 KB)

## Data Availability

The data supporting this study’s findings are available at Data Repository for the U of M (DRUM) and it has been assigned a permanent URL: https://hdl.handle.net/11299/265722.
