# Supplementary figures and images for: Attenuation of Cellular Senescence and Improvement of Osteogenic Differentiation Capacity of Human Liver Stem Cells Using Specific Senomorphic and Senolytic Agents
Source: Stem Cell Rev Rep. 2025 Apr 12;21(5):1523–39. doi: 10.1007/s12015-025-10876-x (PMC12316771; doi:10.1007/s12015-025-10876-x)

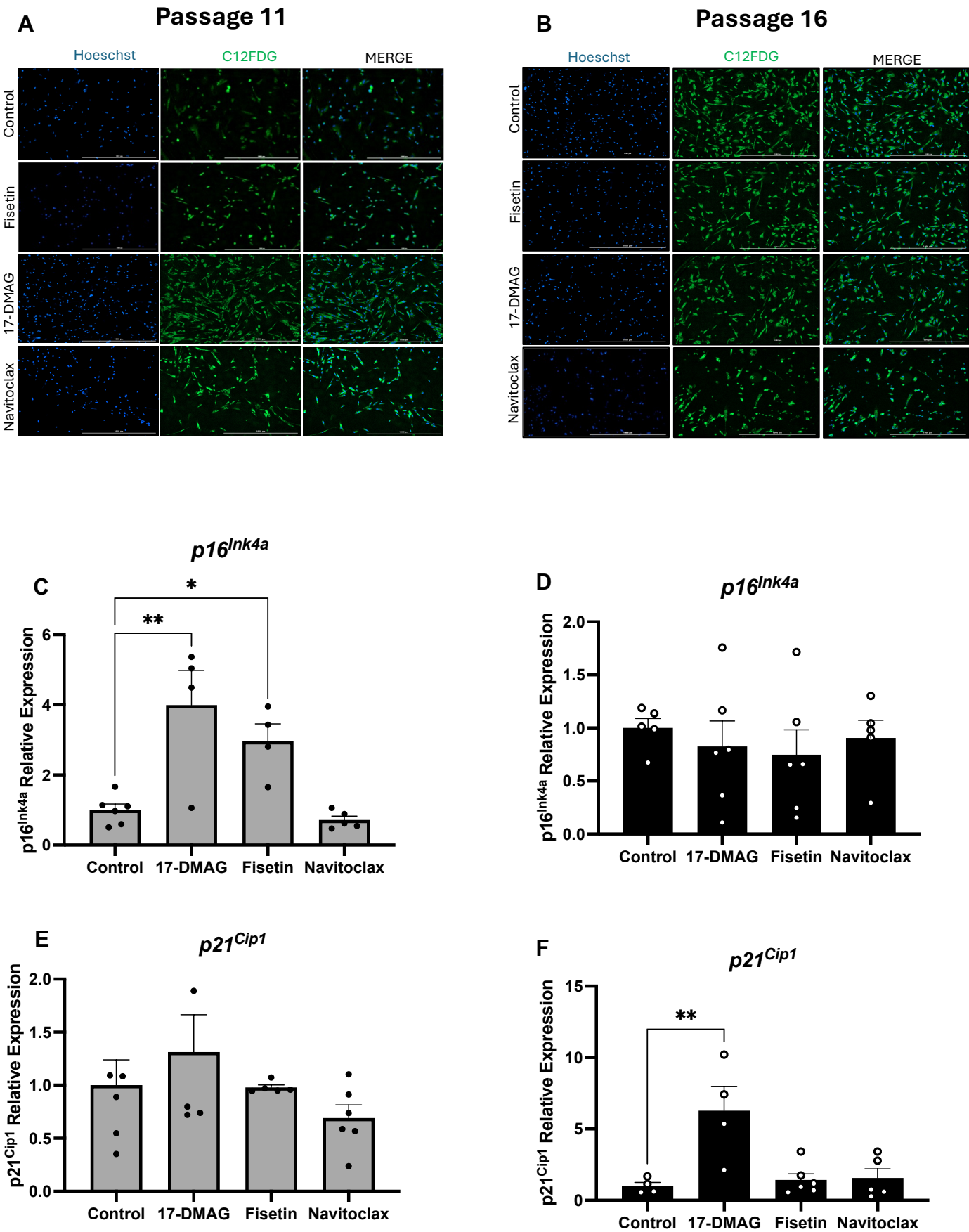

A

## Top 10 KEGG-pathways

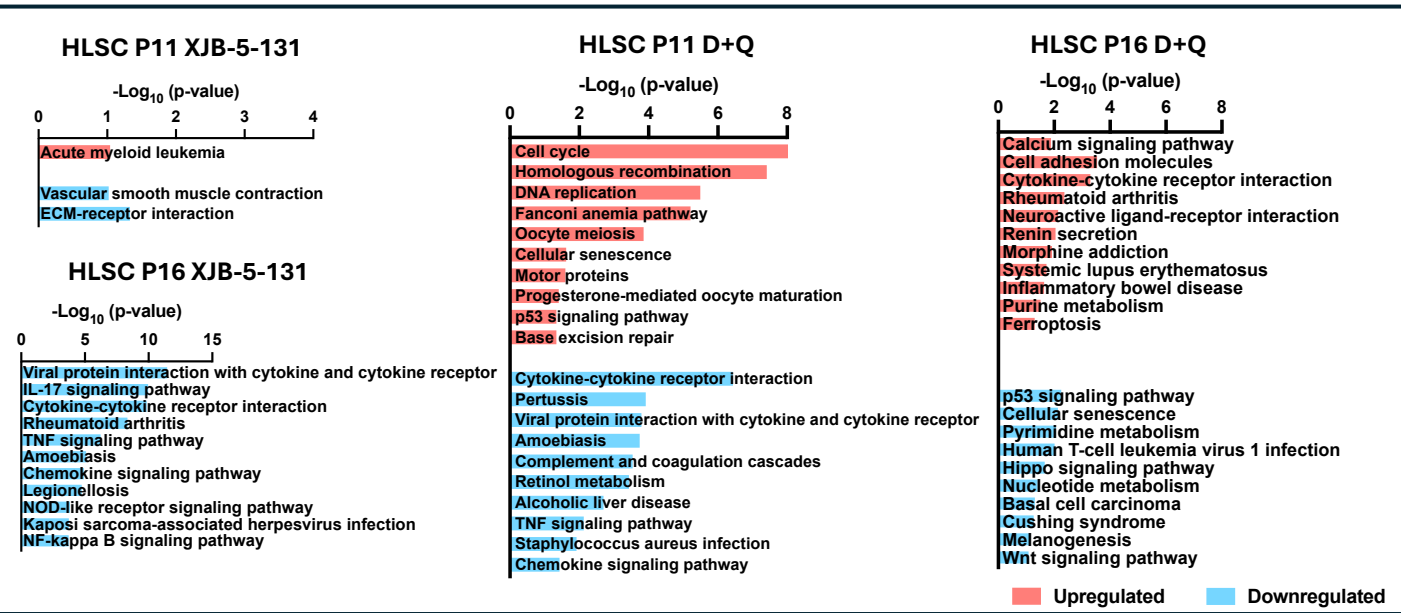

B

## Top 10 Go-terms

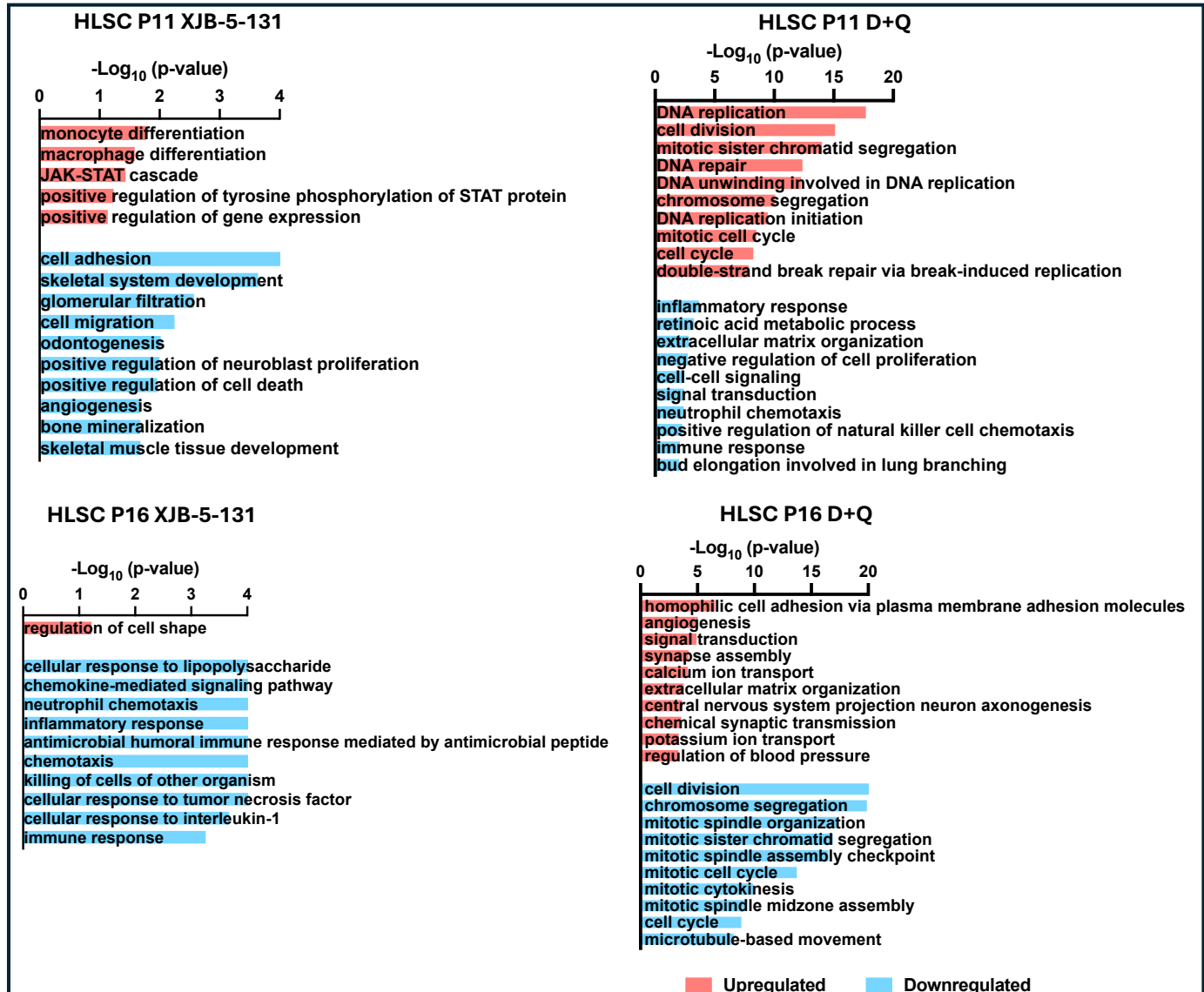

Supplement: Supplementary file 1 — Supplementary file1 (PDF 3642 KB) Supplemental Figure 1. 17-DMAG, Fisetin and Navitoclax did not reduce senescence in late-passage adult human stem cells. (A and B) Expression of the senescence markers p16INK4a and p21CIP1 in HLSCs at passage 11 after 24 hours treatment with Fisetin, 17-DMAg and Navitoclax. (C and D) Expression of the senescence markers p16INK4a and p21CIP1 in HLSCs at passage 16 after 24 hours treatment with Fisetin, 17-DMAG or Navitoclax. All the data are shown as means ± SEM. *p<0.05, **p<0.01 and ****p<0.0001. Supplemental Figure 2. Analysis of the top 10 KEGG pathways and GO terms. (A) Log (p value) analysis of the top 10 KEGG pathways in HLSCs at passages 11 and 16 after XJB-5-131 and D+Q treatment. Blue=upregulated and Red=downregulated. (B) Log (p value) analysis of the top 10 GO terms in HLSCs at passages 11 and 16 after XJB-5-131 and D+Q treatment. Blue=upregulated and Red=downregulated. [file 12015_2025_10876_MOESM1_ESM.pdf]
