# Supplementary material for: Attenuation of Cellular Senescence and Improvement of Osteogenic Differentiation Capacity of Human Liver Stem Cells Using Specific Senomorphic and Senolytic Agents
Source: Stem Cell Rev Rep. 2025 Apr 12;21(5):1523–39. doi: 10.1007/s12015-025-10876-x (PMC12316771; doi:10.1007/s12015-025-10876-x)
Supplement: Supplementary file 2 — Supplementary file2 (DOCX 16 KB) [file 12015_2025_10876_MOESM2_ESM.docx]

**Table S1. mRNA primers used in this study.**

| mRNA | Forward primer | Reverse Primer |
| --- | --- | --- |
| p16^Ink4a^ | CTCGTGCTGATGCTACTGAGGA | GGTCGGCGCAGTTGGGCTCC |
| p21^Cip1^ | AGGTGGACCTGGAGACTCTCAG | TCCTCTTGGAGAAGATCAGCCG |
| IL-8 | GAGAGTGATTGAGAGTGGACCAC | CACAACCCTCTGCACCCAGTTT |
| IL-1β | CCACAGACCTTCCAGGAGAATG | GTGCAGTTCAGTGATCGTACAGG |
| IL-6 | AGACAGCCACTCACCTCTTCAG | TTCTGCCAGTGCCTCTTTGCTG |
| B2M | CCACTGAAAAAGATGAGTATGCCT | CCAATCCAAATGCGGCATCTTCA |
| FGF23 | GGAACAGCTACCACCTGCAGAT | CACCACAAAGCCAGCATCCTCT |
| Osteocalcin | CGCTACCTGTATCAATGGCTGG | CTCCTGAAAGCCGATGTGGTCA |
| RunX2 | CCCAGTATGAGAGTAGGTGTCC | GGGTAAGACTGGTCATAGGACC |
| Col1a1 | GATTCCCTGGACCTAAAGGTGC | AGCCTCTCCATCTTTGCCAGCA |
